# Interpersonal and intrapersonal entrainment of self-paced tapping rate

**DOI:** 10.1371/journal.pone.0220505

**Published:** 2019-07-30

**Authors:** Håvard Lorås, Tore Kristian Aune, Rolf Ingvaldsen, Arve Vorland Pedersen

**Affiliations:** 1 Department of Sport Science & Physical Education, Faculty of Teaching, Art and Culture, Nord University, Levanger, Norway; 2 Faculty of Medicine and Health science, Department of Neuroscience and Human Movement science, NTNU–Norwegian University of Science and Technology, Trondheim, Norway; University of Muenster, GERMANY

## Abstract

Entrainment is a ubiquitous property not only of interacting non-linear dynamical systems but also of human movements. In the study reported here, two premises of entrainment theory were investigated in a tapping task conducted in both interpersonal (i.e. between individuals) and intrapersonal (i.e. between effectors) conditions. Hypothesis 1 was that interacting oscillatory systems should demonstrate synchronisation, which was predicted to emerge as in-phase tapping behaviour in both inter- and intrapersonal conditions. Support for Hypothesis 1 was observed in the in-phase synchronisation of tapping in both individual bimanual trials and uni-manual and bimanual tapping in dyads. By contrast, Hypothesis 2 was that the oscillatory system with the faster initial rate would decelerate, whereas the one with the slower initial rate would accelerate, as manifest in increased self-paced tapping rates amongst participants with initially slower rates and decreased rates amongst ones who initially tapped at faster rates. However, that pattern predicted in Hypothesis 2 was not observed; on the contrary, all participants increased their tapping rates in interpersonal conditions, which occurred significantly amongst participants with the lowest preferred tapping rates. Such an outcome indicates a novel aspect of synchronised movement in humans that warrants further investigation.

## Introduction

In their daily lives, humans tend to synchronise their motor behaviours to those of other people. Examples of the tendency include the synchronisation of gait during side-by-side walking [1,2] and the synchronisation of applause in an audience after a performance [3,4]. That effect, termed *entrainment*, is typically defined as the interaction and consequent temporal alignment of the pace and frequency of two or more persons’ movements (i.e. interpersonal) or the coordination of different effectors within a person (i.e. intrapersonal). Entrainment describes a shared tendency of a wide range of physical systems (e.g. pendulum clocks and pacemakers), as well as biological ones (e.g. fireflies and clouds), involving coordination of temporally structured events via energy transfer and, as such, is an emergent property of physical and biological systems [5–7]. Entrainment is evident in neurologically healthy individuals as well as patients with neurological disorders, for the latter of whom rehabilitation techniques have been designed based upon the concept [8,9].

Derived from physics and dynamical systems theory, a central feature of entrainment is the notion of dynamical systems as non-linear oscillators. According to that concept, dynamical systems can display periodic and repetitive variation, typically temporal in nature, of behaviour around a central value (e.g. a point of equilibrium) or between two or more different behavioural states [10,11]. An example of biological oscillation appears in circadian rhythms, which describe any biological process involving an endogenous oscillation of approximately 24 h. Driven by a circadian clock, those 24-h rhythms have been widely observed in plants, animals, fungi, and bacteria [12,13]. In humans, individual neurons in the central nervous system display rhythmic or repetitive patterns of neural activity, either as oscillations in membrane potential or as rhythmic patterns of action potentials [14,15]. Oscillations in non-linear dynamical systems, however, can exhibit several variations in their period of oscillation (i.e. time to complete one cycle) or in their cyclic frequency (i.e. cycles per unit of time), if not both [16, 17].

Entrainment theory includes two premises regarding the presence of multiple non-linear oscillatory systems in proximity. First, when two or more systems interact, they can assume a common periodicity or frequency, even if they display different periods or frequencies of oscillation while functioning independently [18,19]. That premise stems from the first law of thermodynamics, which states that interacting systems in proximity can exchange energy and, in turn, come to share a similar property [20]. Thus, an essential property of non-linear oscillatory systems is that they always interact [21], which suggests that their behaviour can converge by adjusting their oscillatory rhythm [22]. That effect, called *synchronisation*, appears in various forms of interaction, or *coupling*, between oscillatory systems. Whether synchronisation occurs, however, depends upon the strength of the interaction between the systems (coupling strength), and the degree of detuning or mismatch in frequency between the uncoupled oscillators in their original states [16]. At the same time, even a weak interaction can prompt synchronisation [23].

Synchronisation is generally operationalised in terms of the relative phase between interacting systems and can be distinguished by two synchronous regimes. On the one hand, if two dynamical oscillatory systems move in the same direction and almost simultaneously attain the same positions, then their phases are extremely close and called *in-phase synchronised*. On the other, if the systems move in opposite directions, then they exhibit *anti-phase synchronisation* [16]. The latter form of synchronisation was first described by Christiaan Huygens in 1673, when he discovered that a pair of pendulum clocks hanging from the same support beam had synchronised; not only did their oscillations coincide perfectly, but their pendula moved in opposite directions [24].

The second premise of entrainment theory states that when interacting dynamical systems synchronise, the oscillatory system with the faster initial rate will decelerate, while the one with the slower initial rate will accelerate [25,26]. That supposition derives from principles of dynamic pattern formations, including that a spontaneous cooperative effect emerges when non-linear oscillatory systems interact. Such a spontaneous formation of common periodicity or frequency, termed *self-organisation*, is a process that occurs in many physical, chemical, and biological systems whose original states change [27,28]. From the perspective of synergetics, interactions between dynamical oscillatory systems are subject to control parameters generated by the systems, which upon reaching critical values drive the systems to adopt a new synchronised state following adjustments to their initial oscillations [29,30].

The premise of entrainment theory that the periodicities or frequencies, if not both, of interacting oscillatory systems converge has been widely observed in animal movements, including those of humans. By measuring how movements with different origins coordinate, researchers have demonstrated the clear tendency of those movements to synchronise. Early work on inter-limb phase control by von Holst during the 1930s is considered classical in that respect. In his experiments on the movements of fish fins, for example, von Holst identified two major types of coordination. The first type, which he termed *absolute coordination*, describes a 1:1 correspondence between cyclicities of different structures in which phase and period are the same (i.e. in-phase synchronisation). In the second and far less common type of synchronisation, which he termed *relative coordination*, the movement of the fish fins exhibited different frequencies, although at least one of the phases usually corresponded to that observed during absolute coordination [31,32]. von Holst thus suggested two basic principles that characterise the coordinative properties of oscillators: the maintenance tendency and the magnet effect. *Maintenance tendency*, or the tendency of an oscillator to maintain its rhythm, prompts entirely synchronised movements in a form of absolute coordination. Those steady, rhythmic oscillations work in clear contrast to the *magnet effect*, or the effect that one oscillator exercises over another with a different frequency, such that it seems to magnetically draw and couple the other oscillator to its own frequency [33].

In studies on human movement, the principle of the synchronisation of interacting non-linear oscillatory systems has been widely demonstrated in both intrapersonal (i.e. bimanual) and interpersonal coordination. In tasks in which participants individually move their limbs simultaneously, coordination between the limbs is typically characterised by in-phase synchronisation [34]. In-phase synchronisation is not the only preferred pattern for bimanual coordination, however; anti-phase synchronisation can also be spontaneously adopted, and other phase relations can be learned [35,36]. Similarly, interpersonal coordination, typically between two individuals, is characterised by the in-phase synchronisation of the movements of their limbs. In later studies on the topic, coordination between participants has been examined, for instance, when individuals swing pendula [37], walk side by side on treadmills or overground [38,39], move back and forth in rocking chairs [40], and row on ergometers [41]. Taken together, such studies suggest that even if pairs of people are not instructed to coordinate their movements, their movements become synchronised nonetheless, hence the term *unintentional interpersonal coordination* [42]. Intrapersonal bimanual coordination thus appears to share strong similarities with interpersonal coordination, although bimanual coordination exhibits a higher degree of being phase-locked, whereas interpersonal synchronisation can be more intermittent. With such knowledge, researchers who have directly compared intrapersonal and interpersonal rhythmic bimanual coordination have shown that coupling strength is weaker for interpersonal than intrapersonal coordination, though the coordinative processes nevertheless share striking similarities [43,44]. For example, both types of coordination exhibit comparable transitions between stable patterns of coordination and are similarly affected by changes in the frequency of movement and the detuning of frequency. The same basic dynamical mechanisms thus seem to underlie intrapersonal and interpersonal coordination [45,46]. Previous studies have also tested the co-influence of intra- and interpersonal coordination with systematic manipulations of various task constraints. Here, findings indicate that degree of both interpersonal and intrapersonal coordination can systematically increase due to manipulations of task constraints. However, some experimental manipulations have generated coordination patterns in which suggests that interpersonal coordination can change in the absence of a parallel change in intrapersonal coordination [45,47].

Despite all of those findings, few researchers have directly investigated the other premise of entrainment theory in interpersonal and intrapersonal coordination, in which synchronisation is accompanied by systematic adjustments to rate. Some insights, however, have emerged from studies on how movements are coordinated to external rhythms (i.e. sensorimotor synchronisation) [48,49]. A typical methodological approach applied in those studies is known as the *synchronisation–continuation paradigm*, in which participants are instructed to move in response to a stimulus—they typically tap their index fingers in time with a computer-generated metronome—after which the metronome is switched off, and the participants attempt to maintain the established motor rhythm. The behavioural analysis of that latter phase has been applied to study timekeeping abilities in humans for decades [50]. A common finding regarding the synchronisation–continuation paradigm is a gradual drift in participants’ temporal responses after the stimuli have been removed [51,52]. When performing that task in dyadic (i.e. interpersonal) conditions, participants have tended to not only synchronise their tapping behaviour but also demonstrate larger drifts away from synchronisation while they tap by themselves [53,54]. Those findings from the synchronisation phase might be expected given the instruction to synchronise and maintain the same imposed external rhythm. Meanwhile, the findings from the continuation phase without a metronome, however, suggest that different adjustments to rate can occur in interpersonal compared to intrapersonal timekeeping tasks. Individual differences have also been shown to modulate synchronisation with external auditory rhythms. In such research, participants with intrinsically less stable tapping rates increased their stability, whereas ones with intrinsically more stable rates decreased their stability when spontaneously entrained to external metronomes [55].

However, seeking to demonstrate effects in e.g., the synchronisation–continuation paradigm, such experimental procedures in which various task constraints are imposed have precluded the possibility of analysing both aspects of entrainment—that is, synchronisation and rate adjustments—within the same experimental paradigm. Because participants are required to maintain a specified external rhythm, their observed behavioural responses are always affected by timekeeping processes. Consequently, it remains difficult to disentangle whether, for example, the increased drift in tapping in interpersonal conditions is due to entrainment or simply the resetting of participants’ internal clocks [54]. To study the phenomenon of entrainment, a method arguably needs to be implemented that allows the recording of the same, relatively unconstrained, self-paced movement rate across all conditions. To that end, index finger tapping performed without any specific external stimuli or instruction specifying the rate of movement could be a useful model for behavioural studies concerning entrainment. As a relatively unconstrained voluntary movement, that task in its simplest form involves only alternating back and forth from a single point of contact via the flexion and extension of the finger. The tapping rate that emerges is thus freely chosen or, in other terms, *preferred* or *self*-*paced* by the participant.

Based on all of those considerations, the principal aim of the study reported here was to examine the entrainment of self-paced finger-tapping rates in inter- and intrapersonal conditions. Whereas Hypothesis 1 of the study was that the preferred tapping rate would be synchronised in both inter- and intrapersonal conditions, Hypothesis 2 was that, in interpersonal tapping (i.e. between limbs) and intrapersonal tapping (i.e. between individuals), initially slower tapping rates would increase while initially faster tapping rates would decrease.

## Materials and methods

### Participants

Following approval of the experimental protocol by the Regional Committee for Medical and Health Research Ethics, 12 students (i.e. two men and 10 women) were recruited at the university college in Trondheim. All 12 students were neurologically healthy and had a mean age of 23.8 years (*SD =* 3.4, range: 20–29). Handedness was determined for each participant by means of the Edinburgh Handedness Inventory [56], responses to which were coded on a Likert scale to compute a laterality quotient (LQ). Higher, positive LQs were interpreted as right-hand dominance, and the mean LQ across the sample was 96 (*SD* = 4.0) out of 100, meaning that all participants were right-hand dominant. Participants provided their written informed consent prior to completing the experimental paradigm, and all procedures were performed in accordance with the tenets of the Declaration of Helsinki.

### Apparatus

Finger-tapping tasks were completed with participants sitting comfortably at a table 60 cm in height. Spherical reflective markers, 19 mm in diameter, were attached to the right and left index fingernails to allow the fingertip position to be recorded with six Oqus cameras via a motion-tracking system (Qualisys, Gotëborg, Sweden). Kinematic data were recorded with Qualisys Track Manager 2.4 (Qualisys) at a sampling rate of 300 Hz. The raw kinematic data were exported from the Qualisys Track Manager to MATLAB 7.8 (MathWorks, Natick, MA, USA) for further processing.

### Procedure

**Fig 1:**
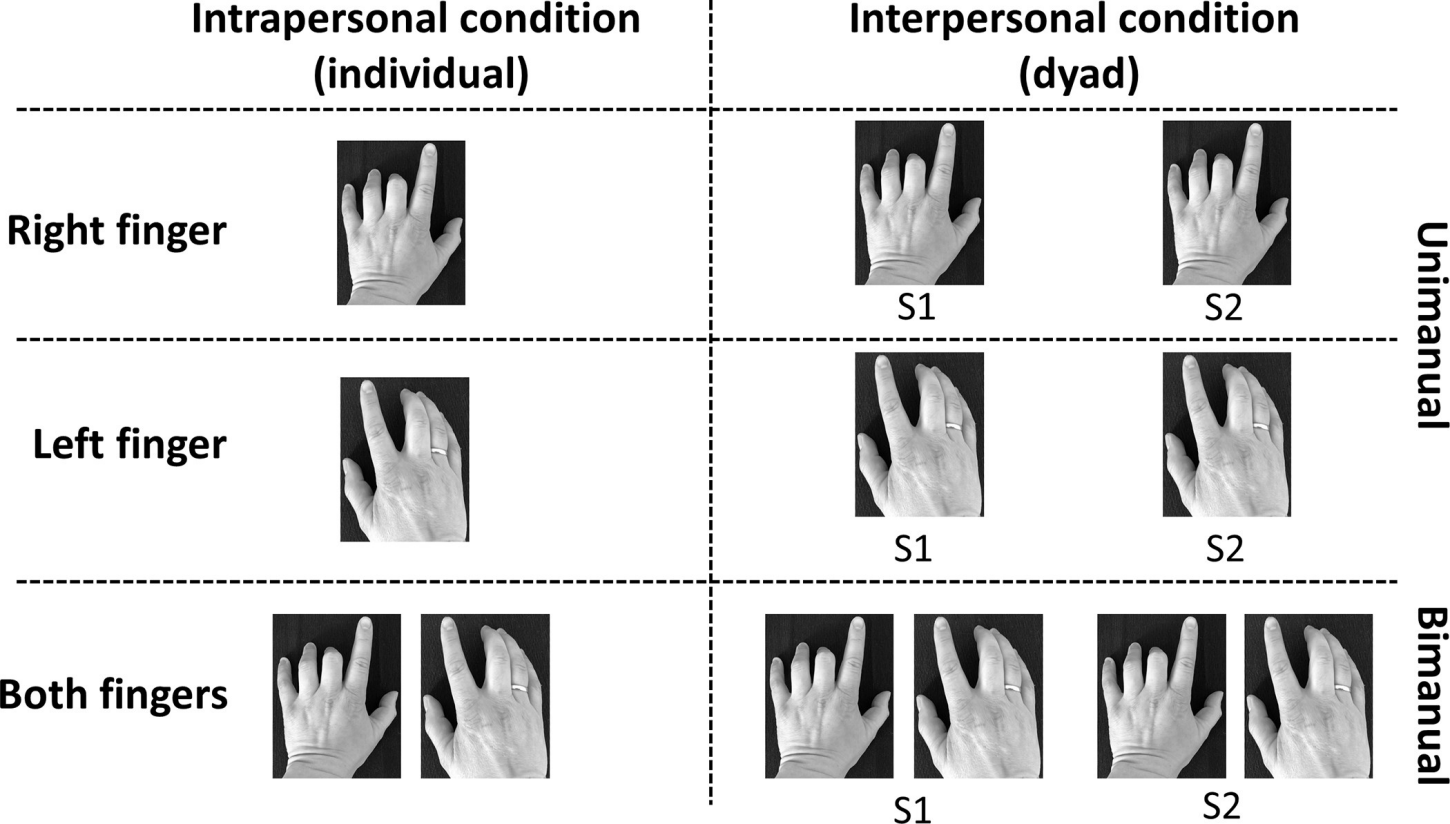
Overview of interpersonal/intrapersonal conditions and tasks. S1 and S2 refers to each subject in dyads.

Three tasks, uni-manual left or right index finger tapping and bimanual (i.e. simultaneously right and left) index finger tapping, were completed under inter- or intrapersonal conditions within a single 45-min session. In adherence with previous studies on self-paced finger tapping, each task consisted of a 3-min trial [57,58], with a 1-min break between tasks. Participants rested their pronated forearms on the table in order to position their hands comfortably next to each other at approximately shoulder-width distance. The order of appearance of the tasks and conditions was counterbalanced. In the interpersonal condition, the tasks were performed with the participants positioned next to each other, which allowed them to both see and hear the tapping of other participants. Thus, all combinations of uni-manual and bimanual finger tapping were measured in both conditions (Fig 1). Participants were carefully informed about the overall aim of the study, but the specific purpose was not revealed in order to avoid any explicit focus on their tapping rate. They were instructed to tap at a comfortable preferred rate in order to produce a steady series of taps (i.e. produce their own constant rhythm). The participants were not given any further instructions about maintaining their preferred tempo or intentionally synchronising with the other participant.

### Data analysis

Raw kinematical data were initially prepared for further processing by means of a Butterworth second-order low pass filter at 5 Hz with a zero phase [59]. To establish the onset of finger taps, a custom algorithm was used to determine all points of a local minima in the z-direction (i.e. superior-to-inferior direction), which corresponded to precise contact with the table and, consequently, points of zero velocity. The preferred rate was thereafter determined by counting the number of local minima throughout the 3-min trials and computed as taps/min.

To examine synchronisation, the Hilbert transform was computed for each finger movements in the z-direction by using the *Hilbert*.*m* function in MATLAB. Next, instantaneous phase values were extracted, and differences in phase angles were computed between the two fingers in the intrapersonal condition or the difference between phase angles of two participants’ fingers in the interpersonal one. In all computations, the relative phase was designated as left to right, and values were computed such that 0 degrees corresponded to in-phase tapping and ±180 degrees corresponded to anti-phase tapping. The mean and variability (*SD*) of the relative phase were calculated using circular (i.e. directional) statistics [60].

### Statistical analysis

To examine Hypothesis 1—that self-paced tapping rate would be synchronised in both inter- and intrapersonal conditions—the overall effect of the two conditions and the three tasks on tapping rate was examined in a within-subject Huyhn–Feldt-corrected repeated-measures ANOVA with partial eta squared as the measure of effect size. The mean and variability of relative phase from the bimanual task were compared between individual and side-by-side tapping with a paired samples *t* test and Hedges’s *g* as the measure of effect size. The latter measure was applied due to the relatively modest sample size.

For Hypothesis 2—that slower initial tapping rates would accelerate while faster initial tapping rates would decelerate—potential differences in changes of tapping rate between intra- and interpersonal tapping tasks amongst participants with slower initial self-paced tapping rates than ones with faster initial self-paced tapping within each dyad were examined by using independent samples *t* tests with Hedges’s *g* as the measure of effect size. To preserve statistical power due to the modest sample size, we considered findings of *p* < .01 to indicate statistical significance. PASW Statistics 25.0.0.1 (IBM, Armonk, NY, USA) was used for all statistical analyses.

## Results

### Tapping rate

**Fig 2:**
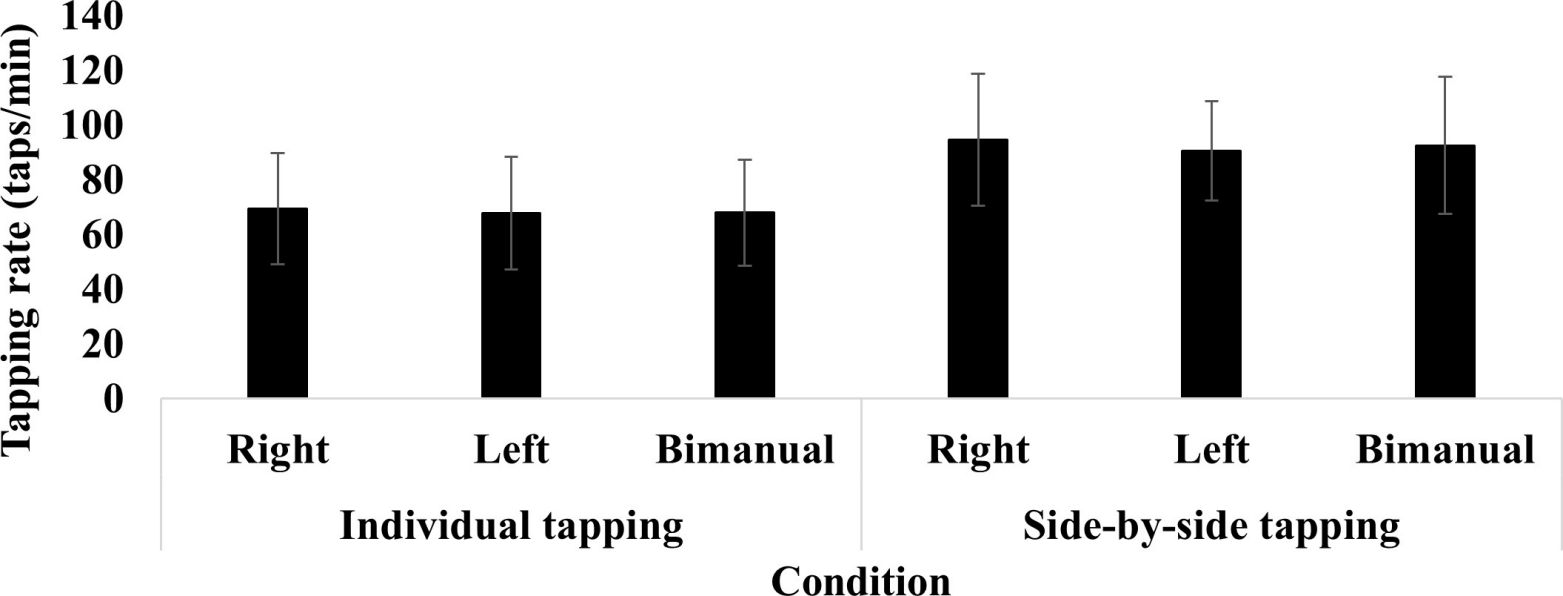
Self-paced tapping rate across hands and conditions. Bars depict mean and error bars depict SD.

**Fig 3:**
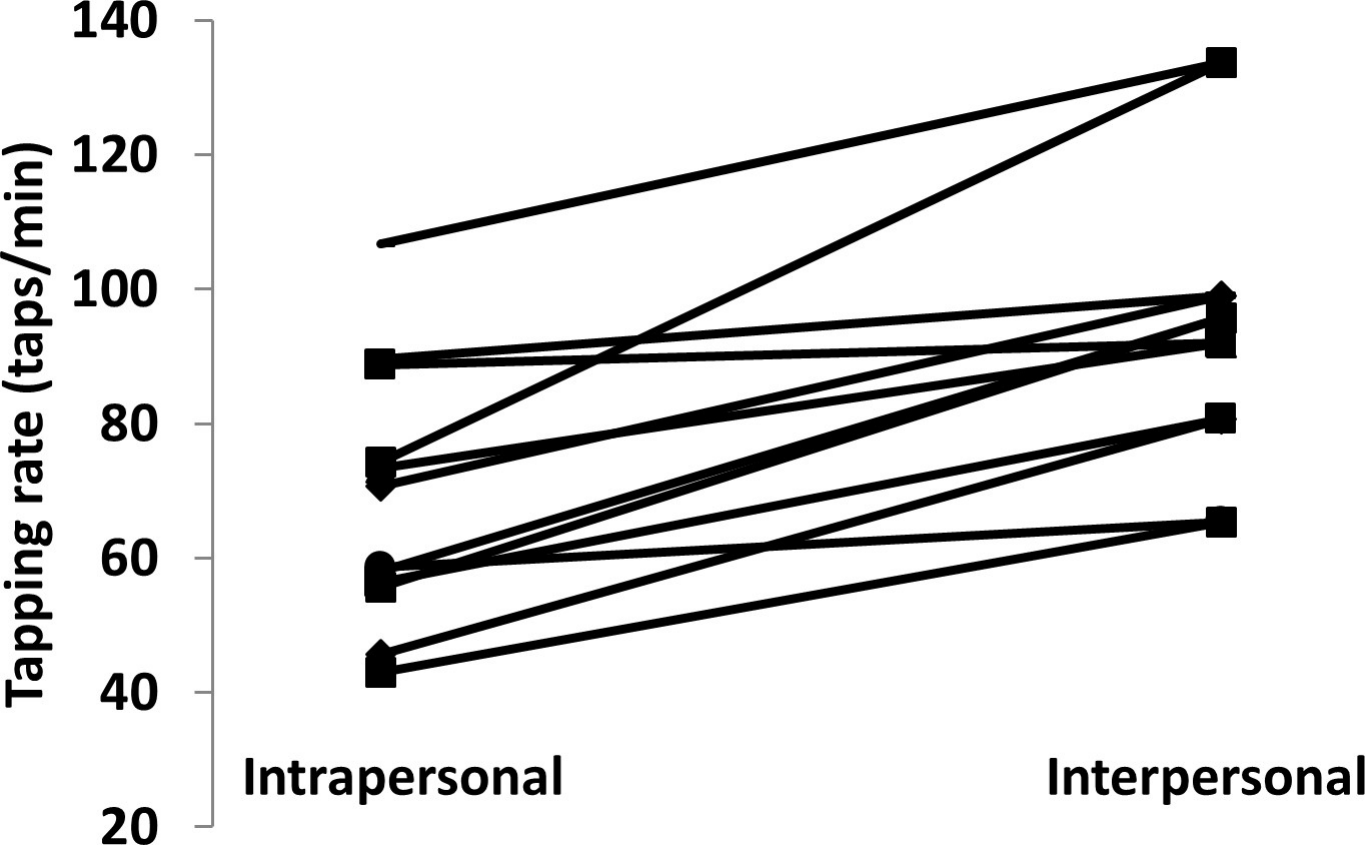
Individual values in self-paced tapping rate from intrapersonal and interpersonal tapping conditions.

**Fig 4:**
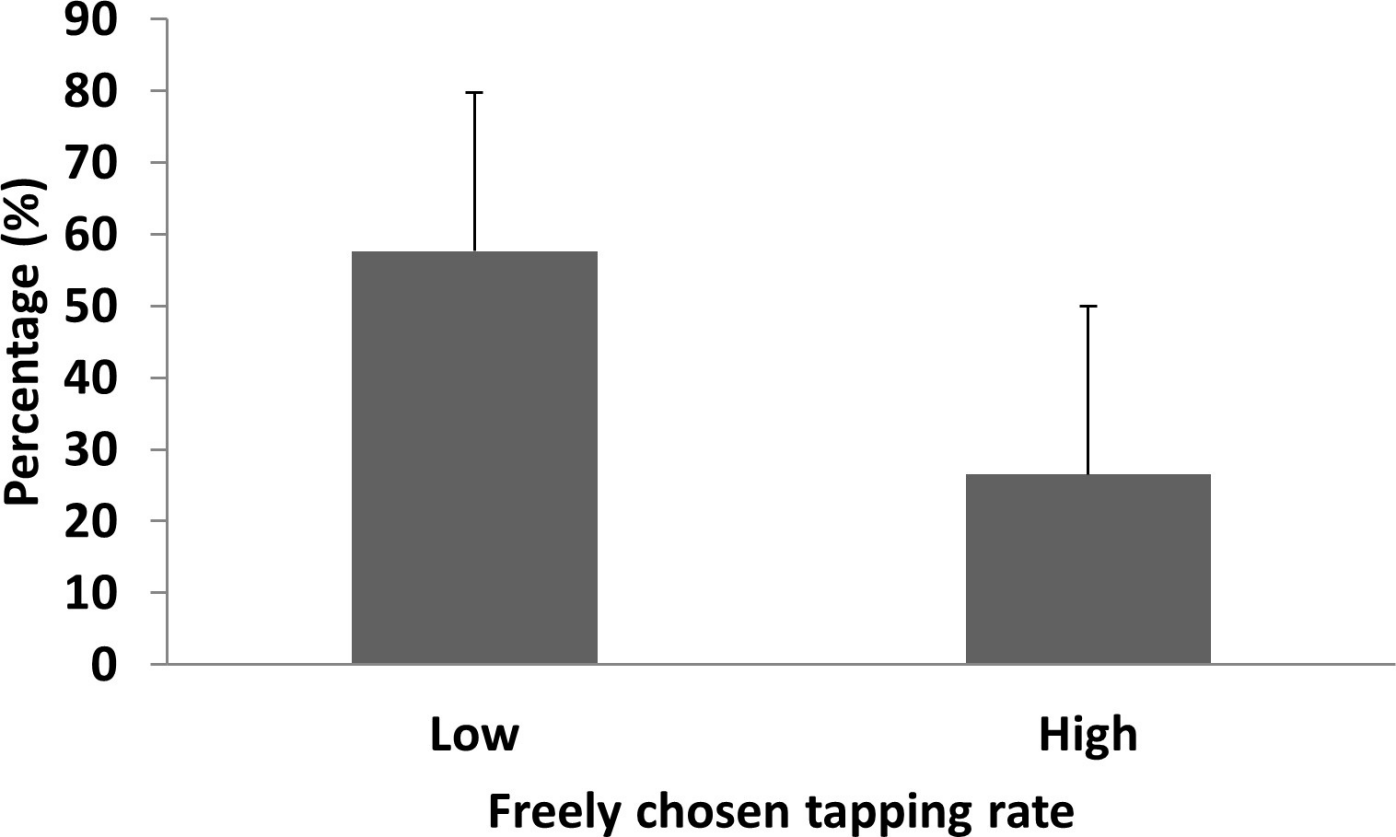
Percent increase in self-paced tapping rate for participants with initial lower preferred rates (‘Low’) and for participants with initial higher preferred tapping rates (‘High’).

Fig 2, which illustrates the overall mean self-paced tapping rates across participants, clearly shows minor differences across uni-manual versus bimanual tapping tasks within the inter- and intrapersonal conditions. Side-by-side tapping, however, seemed to induce higher overall self-paced tapping rates than rates in individual tapping tasks. As clearly visible in Fig 3, all participants increased their self-paced tapping rate from individual trials while tapping in dyads, for an average increase in tapping rate of ~42%. A 2 conditions (inter- vs. intrapersonal) × 3 tasks (uni-manual (right or left) and bimanual) repeated-measures Huynh–Feldt corrected ANOVA performed on mean tapping rate showed the significant effect of the condition (individual vs. side-by-side: *F* (1, 11) = 22.34, *p* < .01, *ηp2* = .67) but no overall significant effect of uni-manual versus bimanual tasks (*F* (1, 11) = 1.01, *p* > .01, *ηp2* = .08). Furthermore, no significant condition × task interaction emerged (*F* (1, 11) = 0.17, *p* > .01, *ηp2* = .02). As shown in Fig 4, participants with slower self-paced tapping rates increased their taps/min significantly more (*t* = 2.37, *df* = 10, *g* = 1.49, *p* < .01) than their partners.

### Relative phase

**Fig 5:**
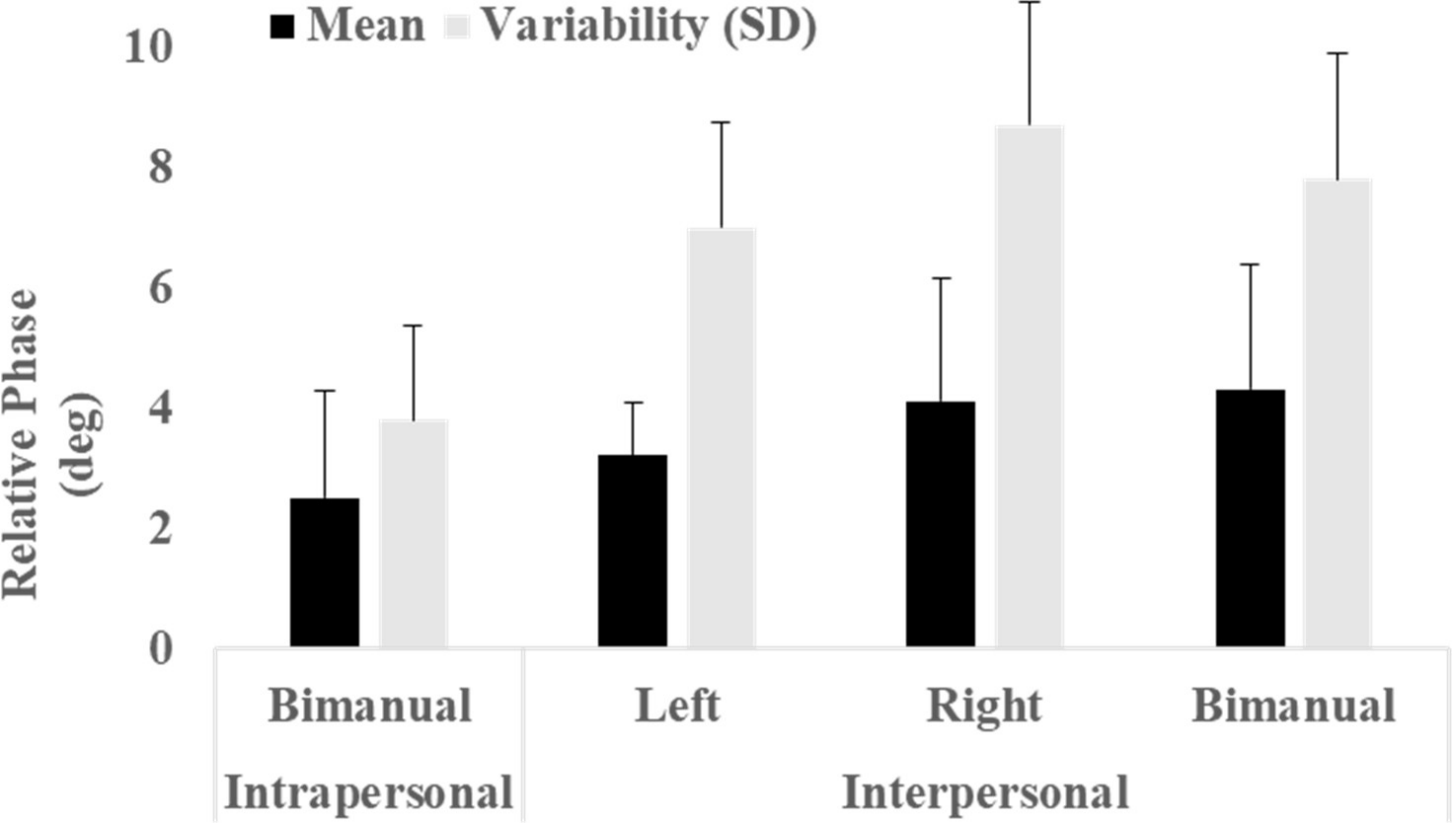
Average mean and variability (SD) of relative phase across hands and conditions. Bars depict mean and error bars SD.

Fig 5 depicts the overall mean and variability of the relative phase across participants. As shown, the relative phase was close to zero in all tasks and conditions. The results of a paired samples *t* test, however, indicated a significant difference between individual bimanual tapping and side-by-side bimanual tapping for the mean of the relative phase (*t* = 2.74, *df* = 11, *g* = 0.89, *p* < .01) but not for variability (*t* = 1.84, *df* = 11, *g* = 0.66, *p* < .01).

## Discussion

The aim of the study was to test two premises of entrainment theory applied to the coordination of inter- and intrapersonal movements, with finger tapping as the behavioural task. The first premise states that interacting systems should demonstrate synchronisation, which in the study was hypothesised to emerge as in-phase tapping behaviour in both inter- and intrapersonal conditions. By contrast, the second premise states that the system with the faster initial rate decelerates, whereas the one with the slower initial rate accelerates; in the study, that supposition was hypothesised to manifest as an increase in self-paced tapping rates amongst participants with slower initial tapping rates and a decrease in participants with initially faster ones. Support for the first premise of entrainment (i.e. Hypothesis 1) surfaced in the in-phase synchronisation of tapping in both individual bimanual trials and uni-manual and bimanual tapping in dyads (Figs 2 and 5). As for the second premise (i.e. Hypothesis 2), no trend emerged such that participants with slower initial preferred tapping rates accelerated their rates or that participants with faster initial rates decelerated theirs. Instead, significant increases in tapping rates from intra- to interpersonal conditions surfaced in all participants (Fig 3), and such increases were significantly greater for participants with the slowest preferred tapping rates (Fig 4).

In line with Hypothesis 1, preferred tapping rates were in-phase synchronised in both inter- and intrapersonal conditions (Fig 5), which lends additional support to the notion that in-phase coordination is a common pattern in entrained human movements [18,34]. Entrainment is postulated to occur because interacting oscillatory systems in proximity exchange energy, following the first law of thermodynamics, and self-organise towards a synchronised state [20,27]. Those basic processes, however, occur via different mechanisms in inter- versus intrapersonal coordination: intrapersonal via neuromuscular coupling in the central nervous system and interpersonal via perceptual processes [45]. Regarding the latter processes, tapping tasks such as the ones used in our study afford potential auditory (i.e. the sound of taps against the table), visual (i.e. observations of the other participant’s fingers), and haptic (i.e. the detection of the table’s movements) perceptual information that might function as possible cues about the partner’s tapping rate.

In the study, the relative phase also approached zero across conditions, which indicates close in-phase synchronisation (Fig 5), although not as much for individual entrainment assessed in the intrapersonal condition. Such results align with the findings of previous studies that compared the stability and strength of coupling in intra- and inter-bimanual coordination [42–46]. In terms of dynamics, the outcome indicates weaker coupling [11,16] in interpersonal entrainment between two motor systems than in intrapersonal entrainment within the same system. That finding should hardly come as a surprise, however, since the interaction between effectors occurs at a faster rate within individuals than the interaction of perceptual information between individuals. Thus, perceptual information regarding another person’s tapping rate might require longer processing than internal feedback processes, which could explain the difference in the relative phases. In either case, the results of our study, as well as of other research, indicates that neuromuscular coupling is stronger than perceptual (e.g. auditory, visual, or haptic) coupling.

Hypothesis 2, concerning the adjustment of rates in entrained movements, expected that participants with faster initial tapping rates would decelerate their rates and that ones with slower initial tapping rates would increase theirs [25,26]. That pattern of results, however, did not surface in either interpersonal or intrapersonal tapping (Fig 3). By contrast, a significant trend emerged in which all participants increased their tapping rates from when they tapped alone to when they tapped with other people, and ones with slower initial preferred rates increased their taps/min significantly more than their partners (Fig 4).

Pinpointing the reason for that latter systematic trend in entrained interpersonal movements is clearly an avenue for further research. Arguably, in-phase entrainment accompanied by an increase in tapping rate, as demonstrated in our study, occurs due to humans’ reliance on predominantly auditory information. In other terms, perceptual dominance on behalf of the auditory system might occur in interpersonal tapping, given that haptic information is processed more slowly than visual and auditory stimuli [61] and because visual perception requires additional effort due to eye fixation and saccades. Since accelerating tapping rate provides more temporal information (i.e. shorter intervals between taps) that the auditory system is especially equipped to process [62], it results in improved temporal control of movements. That notion is in accordance with research on sensorimotor synchronisation, in which taps occur in accordance with metronome beats, and indicates that salient events (e.g. touching a table) occur more precisely and in line with faster rates of stimuli [63]. The phenomenon might also relate to Weber’s law, which holds that changes to shorter intervals between taps are more easily detected than longer ones [64].

The characterisation of the auditory system as the driving force of in-phase interpersonal entrainment has been supported by various studies on auditory distractors during finger tapping, in which movements were strongly attracted to external auditory rhythms [65]. In interpersonal tapping, the taps of co-actors might serve as an auditory attractor compared to self-generated taps, because the former generate perceptual divergence, at least according to the concept of the control parameter in synergetics [29]. Indeed, auditory stimuli have been demonstrated to influence self-paced tapping [66], and self-paced rhythmic movements have been shown to spontaneously entrain with external auditory rhythms, such as those provided by metronomes [54,67]. Consequently, auditory perceptual divergence can function as a control variable that contributes to the self-organising process during in-phase entrainment and the increase of co-actors’ tapping rates as emerging behaviours. In turn, an avenue for further research is to establish whether auditory divergence fulfils the outlined criteria and introduces phase shifts for an order parameter in relation to interpersonal entrainment.

Another possibility for the systematic increase in self-generated tapping rates in entrained movements concerns the potential of increased arousal in social compared to individual contexts. Increased arousal due to the presence of others has been shown to facilitate performance on some tasks [68], and the perceptual information regarding the movements of others and one’s own may energise personal movements [69]. Indeed, social factors have been shown to affect the spontaneous emergence of behavioural synchrony; recordings of movements while participants perform repetitive stepping with a partner who either turned up for the experiment on time or was 15 min late have shown substantially reduced in-phase synchrony when participants interacted with partners who showed up late [70].

The results of our study and of others clearly suggest that motor control of self-paced movements in entrained inter- and intrapersonal coordination result from the relations that exist within and between perceptual–motor systems, not through a specific anatomical or neural mechanism such as a central pattern generator [57,71]. The entrainment of tapping rates can occur independently of the physical coupling of effectors and thus represent an example of the close integration between perceptual processes and motor actions [72]. In-phase coordination appears to be a strong attractor across tasks and movements; individuals and effectors that move simultaneously self-organise towards that tendency as a relatively stable solution within the constraints of the given task.

## Conclusion

The results of our study suggest that in-phase synchronisation, as premised by the concept of entrainment, can emerge in both inter- and intrapersonal tasks, which agrees with the self-paced tapping rates observed, as the relative phase was close to zero across conditions. The second premise of entrainment, however, was not evident in our data; initially slower tapping rates did not increase, and initially higher ones did not decrease in interpersonal or intrapersonal tapping, as was hypothesised. Nevertheless, a systematic trend emerged across participants insofar as they increased their tapping rates from intra- to interpersonal conditions, which was significantly more pronounced for participants with the initially lowest preferred tapping rates. That finding highlights a novel aspect of synchronised movement in humans that deserves further investigation, particularly into the causes and determining processes associated with entrainment.
